# Risk factors for emerging, re-emerging, and newly recognized arboviruses and zoonotic viruses in Brazilian amazon

**DOI:** 10.1371/journal.pntd.0013735

**Published:** 2025-12-23

**Authors:** Livia C. Martins, Jannifer O. Chiang, Lívia M. N. Casseb, Jorge R. de Sousa, Juarez A. S. Quaresma, W. Ian Lipkin, Pedro F. C. Vasconcelos

**Affiliations:** 1 Seção de Arbovirologia e Febres Hemorrágicas, Instituto Evandro Chagas, Ananindeua, Brazil; 2 Núcleo de Medicina Tropical, Universidade Federal do Pará, Belém, Brazil; 3 Departamento de Patologia, Universidade do Estado do Pará, Belém, Brazil; 4 Universidade Federal de São Paulo, Escola Paulista de Medicina, São Paulo, Brazil; 5 Center for Infection and Immunity, Mailman School of Public Health, Columbia University, New York, New York, United States of America; Centers for Disease Control and Prevention, Puerto Rico, UNITED STATES OF AMERICA

## Abstract

Research conducted over seven decades in the Brazilian Amazon has documented the emergence and re-emergence of arboviruses and zoonotic viruses associated with changes in natural ecosystems. Disturbances such as highway construction, deforestation for cattle ranching and soybean cultivation, dam construction for hydroelectric power, intensive mineral extraction, and other human activities can result in outbreaks of viruses maintained in zoonotic or enzootic cycles within forests. These disturbances facilitate the contact between humans and zoonotic hosts or vectors. The potential for a pandemic caused by Amazonian viruses is substantial. The most effective preventative measure is the preservation of the Amazon biome to reduce human encroachment on forested areas, thereby minimizing contact with zoonotic viruses that could initiate a human-vector transmission cycle.

Methods: Search strategy and selection criteria

References for this Review were identified through searches of PubMed with the search terms “Brazilian Amazonia”, “Amazonian Ecosystems”, “Arboviruses” “Emergence and Reemergence of arboviruses”, “Anthropized ecosystem alterations”, “Highway”, “Mining”,”Hydroelectric power plant dams” and “deforestation” from 1974 until April, 2025. Articles were also identified through searches of the authors’ own files. Only papers published in English and Portuguese were reviewed. The final reference list was generated on the basis of originality and relevance to the broad scope of this Review

## Introduction

New studies on the emergence, re-emergence, and identification of new arthropod-borne and zoonotic viral agents are published almost daily [1–3]. Following the emergence of SARS-CoV-2 and the COVID-19 pandemic, speculation has increased regarding the timing and location of the next pandemic [4]. Models suggest that while it could occur anywhere, regions at higher risk include Asia, Africa, and the Americas [5]. A consensus exists that a new virus affecting a naïve population will likely drive the next global outbreak. If an arbovirus is involved, the Amazon biome may be the likely point of origin, with the peri-domestic mosquitoes *Aedes aegypti* and *Aedes albopictus* playing an important role in its maintenance and dispersal, as seen in the last three centuries for dengue (DENV) and yellow fever (YFV), and more recently for Chikungunya (CHIKV) and Zika (ZIKV) [6–10].

### Geography of the Amazon

**Fig 1:**
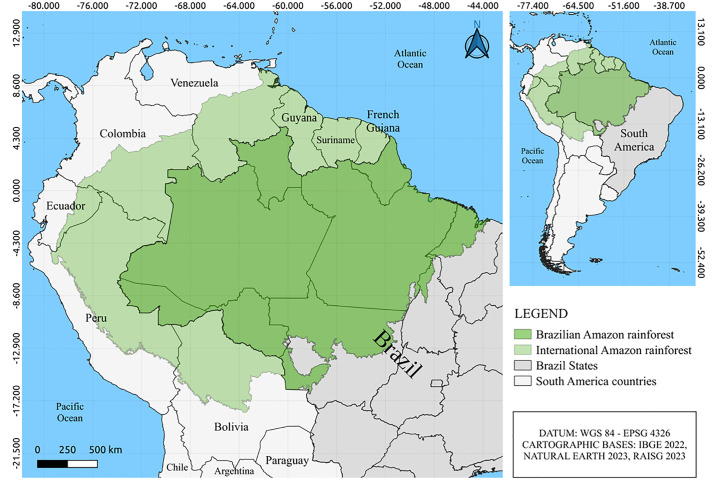
Map of the northern South America showing the Brazilian Amazon region (dark green) and the Amazon (light green) in other countries. The area of Amazon corresponds to around 60% of the Brazilian territory. The base map was obtained from Natural Earth (https://www.naturalearthdata.com), which provides public domain map data freely available for academic and commercial use.

The Amazon spans approximately 50% of South America and encompasses parts of nine countries: Bolivia, Brazil, Colombia, Ecuador, French Guiana, Guyana, Peru, Surinam, and Venezuela. Approximately 60% of the Amazon lies within Brazil (**Fig 1**), with the remainder distributed among the other eight countries [11]. Primarily composed of humid tropical forests, the Amazon is located in northeast South America, bordered by the Caribbean Sea and the Atlantic Ocean to the north, the Brazilian northeast to the east, the Andes mountain range to the west, and the Brazilian cerrado (savannah) and Pantanal wetlands to the south. It is the largest tropical forest in the world. Despite ongoing deforestation, satellite studies estimate that approximately 75% of the original vegetation cover remains intact. In Brazil, the Amazon covers approximately 55% of the country’s territory, extending from the states of Pará and Tocantins in the east to Amazonas and Acre in the west. Approximately 30 million people inhabit this region, with Belém and Manaus being the largest cities, with 1.3 and 2.3 million inhabitants, respectively [11].

In this review, we focus on the Brazilian Amazon, where arboviruses and other zoonotic viruses have been studied for decades.

### Amazon: Biodiversity and the virome

There are approximately 50,000 known vertebrate species, and if we conservatively estimate that each carry 10 endemic yet uncharacterized viruses, the global diversity of viruses could reach more than 500,000.With only approximately 2000 virus species currently identified, we may be underestimating the zoonotic pool by at least 99.8% [12]. Another study estimates the existence of 10^23^ viruses globally [13]. The Amazon has the greatest biodiversity on Earth, in both plant and animal species [14,15], which are essential for the maintenance of thousands of zoonotic and enzootic viruses. The total number of viruses in this region, possibly in the hundreds of millions, forms the Amazonian virome. These viruses persist in various transmission cycles, many of them simple, though the majority are likely maintained in complex cycles [16]. Generally, zoonotic viruses that do not rely on arthropod vectors have simpler transmission cycles or remain unknown due to limited viral isolation data or serological evidence from wild vertebrates that may harbor or have been infected by these viruses [16–18]. However, vector-borne viruses follow more complex cycles because hematophagous insects can transmit viruses across different vertebrate species. Vertebrates may develop short-term viremia, during which other hematophagous insects — either of the same species (primary vector) or different species (secondary vectors) — can become infected through blood meals. After an extrinsic incubation period of a few days to two or three weeks, these insects can pass the virus to new hosts, continuing the viral cycle with or without causing disease [7,19].

**Fig 2:**
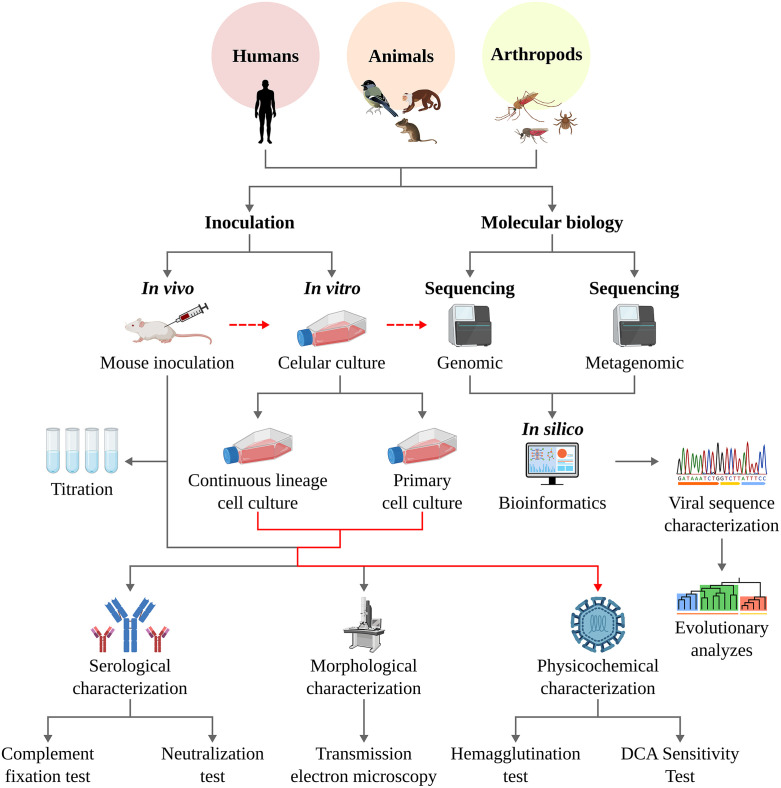
Methods for detecting viral infections from different sources (humans, wild vertebrates, and arthropods). Left side: classical methods for virus isolation and serology. Right side: molecular methods. Fig 2 was entirely generated in R software, using publicly available and open-source packages (e.g., ggplot2, sf, rnaturalearth, rworldmap).

Hundreds of new viruses have been isolated, characterized, and described in the Amazon. For many years, these efforts were restricted to a few laboratories, especially in Brazil, Colombia, and Peru [20–22]. However, the advent of molecular techniques — particularly next-generation nucleotide sequencing — combined with genome and virome studies has expanded viral research. New research centers and institutes, previously uninvolved in viral research due to the complex and costly requirements of virus laboratories, have begun to discover new viruses, mainly through sequencing. These discoveries often include incomplete viral genomes in various hosts, a phenomenon referred to as “virology without viruses” (**Fig 2**) [12,23,24].

The increasing volume of scientific information being published reinforces the need for longitudinal studies to determine the risk factors associated with the emergence or re-emergence of known viruses, as well as previously unknown viral agents. Such studies are also necessary to form hypotheses regarding emergence, transmission mechanisms, and potential control measures [25].

In the following sections, we will analyze the literature on zoonotic viruses, both those transmitted by vectors (arboviruses) and those that are not (zoonotic viruses without arthropod vectors), from a One Health perspective. Furthermore, we provide final considerations on the viruses most likely to re-emerge in the Amazon with the potential to cause major epidemics or even pandemics.

## Risk factors for the emergence/re-emergence of zoonotic viruses and arboviruses

### 1. Amazon biodiversity

The Amazon rainforest is home to the greatest biodiversity of living species on the planet [14,26], with tens of thousands of plant species estimated to be endemic [27]. This vast ecosystem fosters the emergence of life in hundreds of thousands of plant species, facilitating species evolution through natural selection and achieving an unparalleled level of speciation. This extraordinary biodiversity has been maintained in a fragile balance for millennia, probably hundreds of millennia. Any disturbance to this delicate balance results in severe environmental damage [15]. The emergence of viruses in such contexts can be catastrophic [16,28].

Wild vertebrates in the Amazon are believed to number in the thousands of species [29], with many species difficult to identify morphologically. Genetic (chromosomal) analysis is often required to differentiate closely related species that have evolved or are in stages of evolutionary development [30]. However, the greatest animal diversity in the Amazon is that of invertebrates. Thousands of endemic species exist, with a particularly rich diversity among Diptera families, including *Ceratopogonidae* [31], *Culicidae* [8,32], and *Psychodidae* [33,34], all of which are directly associated with the transmission of infectious agents. These families include hundreds of known insect species, and probably many unknown ones, yet to be catalogued. These females are hematophagous (blood-feeding) during their life cycle, specifically to enable egg maturation, thus enabling the gonotrophic cycle, a fundamental step for egg maturation during the replication cycle [7]. In some cases, viral transmission has been demonstrated through vertical mechanisms, as seen with DENV and YFV [7,30].

Hematophagous insects, alongside wild vertebrates — particularly birds and mammals, and to a lesser extent, reptiles, amphibians, and possibly fish — constitute essential elements for maintaining viruses biologically transmitted by insects in the forest. These are known as arboviruses, a term derived from “arthropod-borne viruses” [35].

### 2. Maintenance cycles

Zoonotic viruses that lack an arthropod vector depend on a specific species (primary host) for their maintenance and transmission to other vertebrate species (secondary hosts). Their maintenance cycles are generally simple, involving few species, often from a single family. Classic examples include Arenaviruses (family *Arenaviridae*) and Hantaviruses (family *Hantaviridae*; order *Bunyavirales*) [17,18,36,37]. Practically all known arenaviruses and hantaviruses infect rodents, particularly those from *Murinae* and *Sigmondontinae* subfamilies of the *Muridae* family, as their primary hosts. However, exceptions exist, as viral isolation and serological evidence suggest that bats may also serve as primary hosts for some hantaviruses, though these are typically not pathogenic to humans or other vertebrates [36].

Notably, rodents and bats, the primary hosts of hantaviruses, and rodents for arenaviruses, do not exhibit clinical symptoms, even when highly infected [28,37]. The mechanisms behind this asymptomatic state are not yet fully understood but likely involve immune evasion strategies that result in immunological tolerance, allowing persistent or perennial infections. This commensal relationship is essential for both virus and host survival, a pattern seen in other zoonotic viruses such as paramyxoviruses and influenza viruses, and their primary hosts [38–41]. Detailed molecular and cellular studies further support this, indicating that the processing of MHC class I molecules, which facilitate CD8 + T cell recognition, is associated with viruses that require intracellular replication. In contrast, MHC class II molecules, which are primarily responsible for CD4 + /B cell responses, are associated with organisms that replicate extracellularly [38,42]. In contrast, some zoonotic viruses, such as the rabies virus, cause disease in primary hosts such as bats, carnivores, and non-human primates [43].

The case of Influenza A viruses is more complex. These viruses can infect wild and domestic birds, pigs, and humans. Due to their segmented RNA genome, the combination of the two main genes, which encode the hemagglutinin (H) and neuraminidase (N) proteins, can generate highly virulent strains capable of causing severe clinical outcomes or widespread dissemination, potentially resulting in a pandemic. Point mutations, antigenic drift, and antigenic shift further contribute to the pathogenesis of more aggressive strains [44,45].

The evolutionary relationships between arboviruses and their arthropod vectors are intricate. In the Brazilian Amazon, research conducted for more than 70 years by four generations of scientists showed the vast complexity in the maintenance cycles of most of the isolated arboviruses. Virological and serological data from these studies allowed some of the researchers to propose their maintenance and transmission cycles [19,46,47].

A well-known example of such a cycle involves the Mayaro virus (MAYV) (family: Togaviridae; genus: *Alphavirus*). Originally isolated in Trinidad and Tobago from a cluster of febrile illnesses, MAYV has been identified as the cause of outbreaks of febrile illness accompanied by rash and arthralgias [48–50] in the pan-Amazon region. Cases and outbreaks have been reported in Bolivia, Brazil, Colombia, French Guiana, and Peru, with strong serological evidence of infection in other Amazonian countries [49,50]. Outbreaks and epidemics of MAYV have occurred in small, forest-adjacent communities and villages, occasionally in areas recently deforested for planting extractive agriculture, pastures, or even settlements for housing and family farming. MAYV is frequently isolated from *Haemagogus* mosquitoes, particularly *Haemagogus janthinomys*, which are primarily forest-dwelling and rarely travel from forest edges [16,40,50]. This species inhabits the forest canopy and bites nonhuman primates, arboreal rodents, and wild birds. Consolidated data from the Brazilian Amazon indicate that MAYV is maintained in a primary cycle among *Haemagogus* sp. and non-human primates, from which it has been periodically isolated. Although MAYV has not been isolated from the blood or tissues of birds and arboreal rodents, serological evidence frequently shows antibodies specific to MAYV in their plasma, making them candidates as secondary hosts [51].

Notably, the same species of mosquito can transmit multiple arboviruses, and a single arbovirus can be transmitted by several species of hematophagous mosquitoes. This likely serves as an evolutionary strategy to ensure viral survival in nature [19,47]. *Haemagogus janthinomys* mosquitoes are not only the primary vectors of MAYV but have also been isolated and implicated as the main transmitter of the yellow fever virus (YFV) in a primary cycle involving non-human primates occurring in forest treetops [52]. Other *Haemagogus* species have provided YFV isolates in the Amazon, making them candidates for secondary vectors [53]. In southern Brazil, southwestern South America, and the southern cone region, *Haemagogus leucocelaenus* has been identified as the main vector of YFV in the primary cycle, while other species play secondary roles in YFV transmission [52,54,55]. Similarly, *Sabethes* sp., though less frequently associated with YFV than *Haemagogus,* have also been identified as secondary vectors [53]. In rare situations, YFV has been isolated solely in the genus *Sabethes* [56].

### 3. Viral tropism and transmission

**Fig 3:**
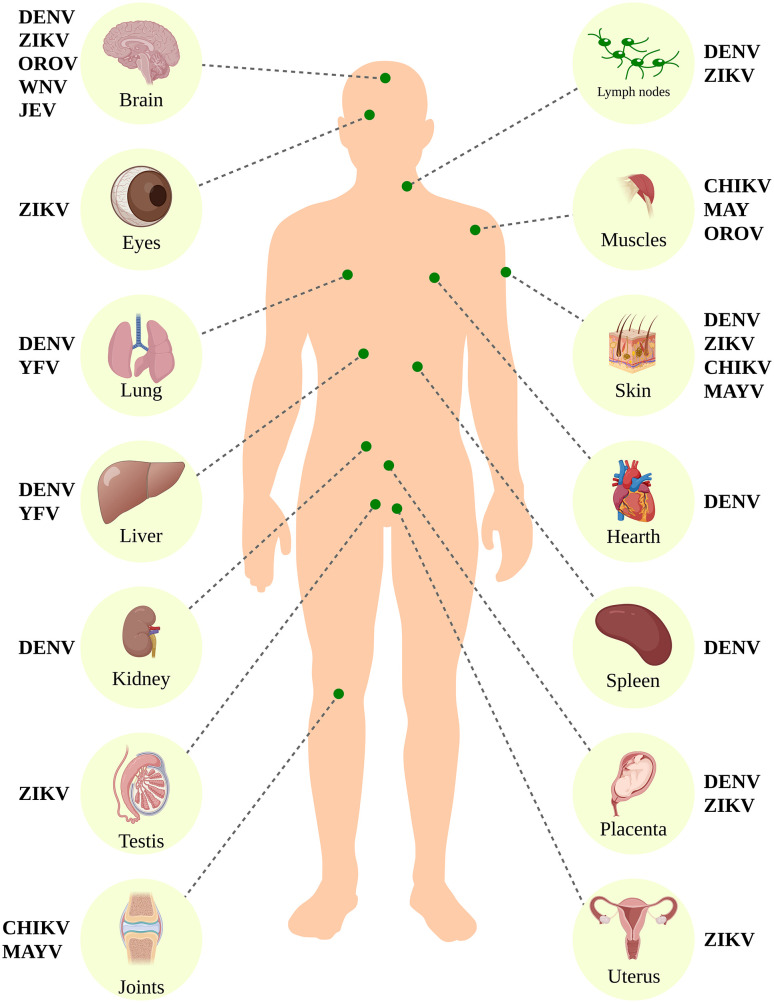
Biological specimens used for diagnosis of arbovirus infection and examples of arboviruses frequently detected in these specimens. CHIKV (Chikungunya virus), DENV (Dengue virus), JEV (Japanese encephalitis virus), MAYV (Mayaro vírus), OROV (Oropouche virus), WNV (West Nile virus), YFV (Yellow Fever virus), and ZIKV (Zika virus). Fig 3 was entirely generated in R software, using publicly available and open-source packages (e.g., ggplot2, sf, rnaturalearth, rworldmap).

For arboviruses to successfully infect vertebrate hosts, they must induce high-titer viremia and systemic infection (**Fig 3**). In general, viruses that cause systemic infections replicate more efficiently in some organs or tissues more than others. This can occur for several reasons, including the affinity of viral antigenic determinants, cell surface receptors, or their interaction [57].

Some viruses have identical entry and exit (transmission) ports, such as respiratory tract viruses like influenza and SARS-CoV-2, which are more easily transmitted and do not require arthropod vectors for transmission [41]. Others, for example enteroviruses, rotaviruses and noroviruses, enter orally and exit via feces to initiate new cycles of food and water borne infection [58,59]. The tropisms of these two groups of viruses, respiratory and gastrointestinal, are intrinsically related to the adaptation by these viruses to the cellular receptors of the epithelial cells of the organs that make up the respiratory and gastrointestinal tracts, respectively [45,59].

The entry point for arboviruses is almost always the bite of infected hematophagous insects. After an infectious bite, the viruses are received in the dermis by DC-SIGN+ dendritic cells or local macrophages and carried to regional lymph nodes, where they undergo initial replication. After replicating in the lymph nodes, arboviruses enter the lymphatic circulation, reach the bloodstream, and are transported throughout the body [60–62]. Arboviruses predominantly infect organs with high receptivity and show different tropisms. Viruses from the same family and genus, such as flaviviruses (e.g., Flaviviridae *Flavivirus*), have different tropisms. It is interesting to note that the flaviviruses predominantly transmitted by mosquitoes of the *Culex* genus have neurotropism and generally cause encephalitis with greater or lesser severity, as is the case with the viruses JEV, SLEV, ROCV, WNV, etc., transmitted by mosquitoes species of the *Culex* genus, whereas the flaviviruses predominantly transmitted by mosquitoes of the genus *Aedes* are hepatotropic and are more frequently associated with hemorrhagic fevers, such as YFV and DENV; they can also severely affect the lungs, kidneys, and heart by causing systemic damage to organisms [52,61,62] (**Fig 3**). ZIKV is situated in an intermediate position. Indeed, it is horizontally transmitted by *Aedes aegypti* but also vertically during pregnancy and is associated with severe encephalitis in neonates with severe congenital malformations, including microcephaly and arthrogryposis [63,64].

### 4. Anthropogenic perturbations of amazonian ecosystems and the emergence of viral threats

**Fig 4:**
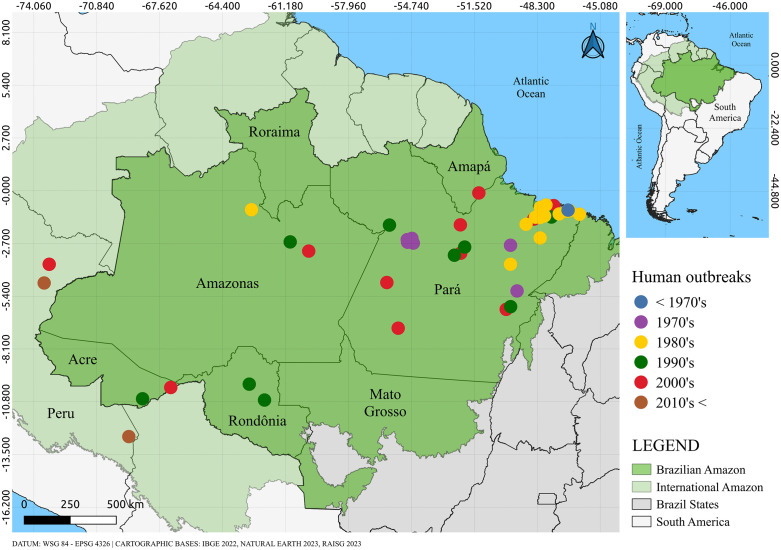
Locations of Oropouche fever epidemics in northeastern South America. The base map was obtained from Natural Earth (https://www.naturalearthdata.com), which provides public domain map data freely available for academic and commercial use.

Human actions in natural ecosystems play important causal roles in the emergence and re-emergence of viruses, particularly arboviruses [16]. A well-known example of this mechanism is OROV, which has always emerged in recently occupied areas in the Amazon, where the vector, the midge *Culicoides paraensis*, takes advantage of decomposing organic remains to reproduce. When OROV emerges inside this population, typically in immigrants, it causes Oropouche fever, a febrile viral condition that can in certain cases result in meningitis or meningo-encephalitis [20,21,28]. OROV has recently spread throughout Brazil [65] and reached other countries in Latin American and the Caribbean including Cuba [66]. In Brazil it has been associated with infections and disease during the pregnancy resulting in microcephaly and other congenital anomalies, as well as miscarriage, stillbirth, and neonatal deaths [67]. OROV epidemics have had an impact on Amazonian populations, with epidemics occurring in the last 53 years in urban centers and newly occupied areas, with an estimated burden of more than 500 thousand cases [68] (**Fig 4**).

Deforestation for agricultural activities, such as cattle raising and soybean planting is also associated with the emergence of new viruses or the re-emergence of known viruses [16,69]. The arboviruses most frequently associated with these activities are YFV, OROV, and MAY, but may include novel viruses [16].

The construction of hydroelectric plants and their reservoir lakes and intensive mining has also contributed to the explosive increase in the population of arthropod vectors, facilitating the emergence of new viruses [8,16,70]. An example is the construction of the Tucuruí hydroelectric plant (UHE-TUC) that led to the emergence of dozens of new viruses as well as the emergence of known but only sporadically isolated arboviruses [70]. The damming of the waters of the Tocantins River to form the UHE-TUC reservoir lake resulted in the accumulation of immense numbers of phlebotomous insects that subsequently invaded the urban areas of Tucuruí, Novo Breu Branco, Novo Repartimento, and other municipalities close to UHE-TUC. The captured insects provided dozens of new arbovirus isolates from the Changuinola group (Sedoreoviridae, *Orbivirus*), Anopheles A, and Gamboa groups (Peribunyaviridae, *Orthobunyavirus*). Gamboa virus (GAMV), a previously undescribed virus in the Brazilian Amazon, is transmitted by the mosquito *Aedeomyia squamipennis*, with wild birds as its primary hosts; however, there are no descriptions of infection in humans, which was probably taken to the UHE-TUC by migratory birds. Several other new arboviruses have been isolated, mainly from hematophagous insects, but their potential to cause diseases in humans and domestic and production animals is unknown [16].

**Fig 5:**
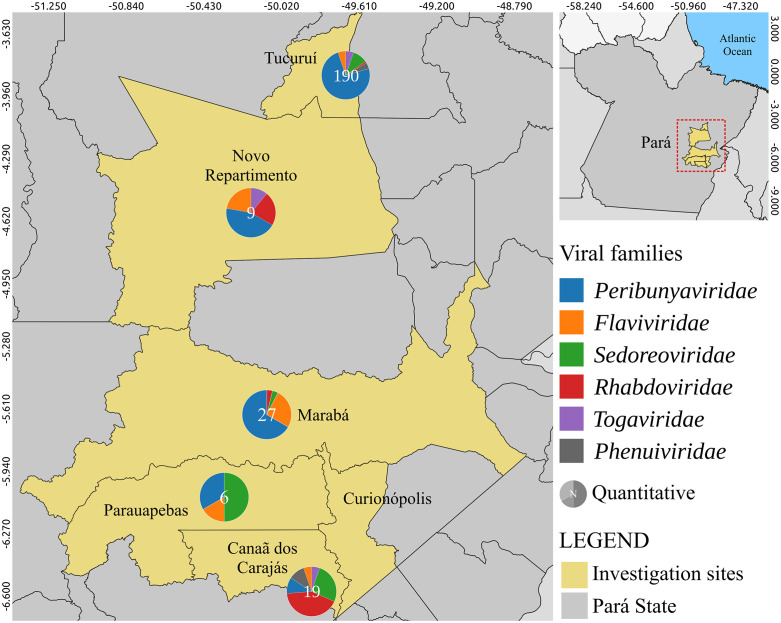
Map of the Serra dos Carajas region where the Carajás mineral province is located in the southeast of the State of Pará, with number of virus isolates obtained according to the virus family and by municipality of study. The base map was obtained from Natural Earth (https://www.naturalearthdata.com), which provides public domain map data freely available for academic and commercial use.

**Fig 6:**
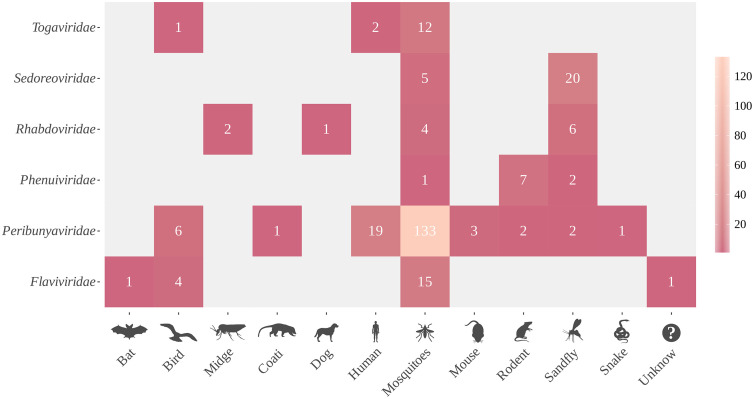
Animal sources for isolation of new or emerging/re-emerging viruses and their classification into viral families in the Brazilian Amazon. Fig 6 was entirely generated in R software, using publicly available and open-source packages (e.g., ggplot2, sf, rnaturalearth, rworldmap).

The Serra de Carajás is an immense region in the state of Pará, involving at least six municipalities (**Fig 5**). Iron mining is the main activity in this mineral complex; however, gold, copper and other ores have also been extracted. More than 30 years of studies in proximity to four mines in this area by the IEC, has enabled isolation of dozens of new viruses from phlebotomous insects, wild vertebrates, mainly birds, and mammals (especially rodents), but also from humans exposed to both wild and urban vectors. Outbreaks of dengue, Oropouche, Chikungunya, and Mayaro fevers have also been reported there [71,72]. In addition, several previously unknown arboviruses have been isolated in the Carajás region, notably new members of the VSV serological group (Rhabdoviridae, *Vesiculovirus*), Phlebovirus group (Phleboviridae, *Phlebovirus*), and Changuinola (Reoviridae, *Orbivirus*) [22,46,72]. **Fig 6** shows the isolation of viruses from different families obtained from different sources, demonstrating the biodiversity of source biological specimens for obtaining new and emerging/re-emerging viruses in the Carajás region of the Brazilian Amazon.

Finally, over the last 60 years, several highways have been opened in the Brazilian Amazon and communities have been established along them, such as Belém-Brasília, Cuiabá-Santarém, Manaus-Boa Vista, Manaus-Porto Velho, and Transamazônica [28,73,74]. Studies conducted by the IEC in some of them showed that these highways were accompanied by secondary roads that allowed the settlement of thousands of migrants who were encouraged to occupy the “housing void in the Amazon,” an initiative of military governments in the 1960s until the beginning of the 2000s, with the slogan “integrate so as not to deliver.” They opened several highways and dozens of side roads on each highway, which accelerated deforestation. Many microbial agents that were not previously known in the Amazon have begun to circulate, mainly parasites and viruses. Many new and emerging or re-emerging arboviruses have been isolated and characterized from humans, wild vertebrates, and arthropods. Consolidated data from these studies have been published in several articles [16,28,73,74]. They have also revealed the emergence of new arboviruses and flexal arenaviruses (Arenaviridae, *Arenavirus*).

## Future perspectives

The expansion of cattle and soy industries in the Amazon Basin has increased deforestation rates and will soon push all-weather highways into the core of the region. A comprehensive conservation strategy is urgently needed to protect the Amazon Basin, its watersheds, the full range of species and ecosystem diversity, and the stability of the regional climate [75]. Although this will be politically and economically challenging, there is still time to mitigate risk if we can commit to containing deforestation and to supporting initiatives to reduce climate change. Failure to do so will result in the emergence of as yet unknown viruses and the re-emergence of endemic viruses with catastrophic consequences as exemplified by the recent case of the emergence of Oropouche fever in South and Central America and the Caribbean [66], and the occurrence of microcephaly and other congenital malformations [67]. These recent outbreaks highlight the importance and urgency of investing in comprehensive viral surveillance in the Amazonian ecosystem and in other biomes in South American countries.

## Limitation of study

Although the studies were conducted over a period of 70 years, generally, with rare exceptions, they were not conducted in the same areas, which certainly significantly limits the conclusions presented here. Furthermore, the studies were conducted primarily due to public health demands, such as outbreaks or epidemics, or due to the development of economic activities such as the construction of hydroelectric plants, mineral exploration, etc., or to improve mobility structures in the lives of Amazonian populations, such as the opening of highways. With the exception of the Serra de Carajás region, where the IEC has been conducting longitudinal studies for over 30 years, in many of the other study areas, there was no systematic continuity in the studies or they were interrupted after a maximum of five years, which also limits the focal rather than longitudinal findings in these areas.

## Conclusion

Over the course of 70 years of studies by the IEC on arboviruses and zoonotic viruses, it has become clear that disturbances in Amazonian ecosystems result in the emergence of new enzootic viruses and the re-emergence of known viral agents that cause epidemic diseases in humans, such as CHIKV, DENV, MAYV, OROV, YFV, and ZIKV. These agents can result in outbreaks not only in human populations resident in the Amazon, but also to urban populations in Brazil and beyond. Global air travel can facilitate rapid dispersion of vectors that carry DENV [21], CHIKV [9], ZIKV [10], as well as human infected with respiratory pathogens like SARS-CoV-2 [76]. As Shope noted in 1997, “it is safe to say that most emerging arbovirus diseases follow ecological modifications. It is naïve to think that humans will stop building cities and dams or stop entering and destroying the forest. We can, however, learn more about risk and risk management, and we must continue to support environmental and health assessments and begin to believe our scientifically based predictions and act on them.” [69].
